# Knowledge, attitudes and behaviors of Latinas in cleaning occupations in northern New Jersey: a cross-sectional mixed methods study

**DOI:** 10.1186/s12995-021-00343-x

**Published:** 2021-12-06

**Authors:** Erin Speiser, Genevieve Pinto Zipp, Deborah A. DeLuca, Ana Paula Cupertino, Evelyn Arana-Chicas, Elli Gourna Paleoudis, Benjamin Kligler, Francisco Cartujano-Barrera

**Affiliations:** 1 The Deirdre Imus Environmental Health Center®, Hackensack University Medical Center, Hackensack, NJ USA; 2 Department of Interprofessional Health Sciences & Health Administration, School of Health and Medical Sciences, Seton Hall University, Nutley, NJ USA; 3 James P. Wilmot Cancer Institute, University of Rochester Medical Center, Rochester, NY USA; 4 Office of Research Administration, Hackensack Meridian Health, Edison, NJ USA

**Keywords:** Environmental health, Occupational exposures, Cleaning, Latina, Population health, Environmental justice, Health disparities

## Abstract

**Background:**

In the United States, 88.3% of all 1,163,000 maids and housekeeping cleaners are female, and approximately half of them Latinas. Latinas are understudied and underrepresented in health research, particularly involving chemical exposure in cleaning practices, lack of job training, and inadequate access to personal protective equipment. The purpose of this study is twofold: 1) to examine the knowledge (via training experiences), attitudes and behaviors of a heterogeneous group of Latinas who clean occupationally and 2) to assess their cleaning practices at work and at home.

**Methods:**

This mixed-method study consisted of two phases: 1) three focus groups to explore knowledge (via training experiences), attitudes, and behaviors regarding cleaning practices (*N* = 15) and 2) a 43-question cross-sectional survey. Focus group audio recordings were analyzed using descriptive and in vivo coding and then coded inductively to explore thematic analysis. Statistical analysis of the survey evaluated means, frequency and percentage for each of the responses.

**Results:**

Participants (*n* = 9) were women (mean age = 48.78 and SD = 6.72) from South America (*n* = 5), Mexico (*n* = 1), El Salvador (*n* = 1) and Dominican Republic (*n* = 2). The mean length of time living in the US was 18.78 years and over half (55.6%) worked in the cleaning industry for 10 or more years.

Findings from the three focus groups (*n* = 15) included that training in cleaning often occurred informally at a very young age at home. Participants reported cleaning in groups where tasks are rotated and/or shared. Most were the primary person cleaning at home, suggesting increased exposure. Gloves and masks were the most frequently used PPE, but use was not consistent. For participants who purchase their own products, driving factors included price, smell and efficacy. Some participants used products supplied or preferred by the employer.

**Conclusions:**

Latinas in cleaning occupations face a range of social and health barriers including lack of safety and health training, inadequate PPE and low literacy. To address these issues, the development of an intervention is warranted to provide training and resources for this critical population of essential workers.

## Background

### Latinas in occupational cleaning: a population health perspective

In the United States, 88.3% of all 1,163,000 maids and housekeeping cleaners are female, and of those, 46.1% are Latinas or Hispanic [1]. Many are immigrants and employed informally, within private homes, as well as in restaurants, hotels, and other locations [2]. According to the U.S. Department of Labor, “Duties may include making beds, replenishing linens, cleaning rooms and halls, and vacuuming” [3].

While the current Latino population appears relatively young and healthy, the combination of population growth, environmental exposures, aging, and other factors are expected to contribute to declining health in coming decades [4]. In particular, incidence of invasive cancers among U.S. Latinos is projected to increase 142% by 2030 [5]. Therefore, studying the population health of cleaning professionals and occupational chemical exposure means engaging an understudied population which faces multiple social barriers and health challenges [2, 6–19].

### Occupational health and worker rights

In the U.S., the Occupational Safety and Health Administration (OSHA), in the Department of Labor, protects worker rights, including cleaning professionals. However, many of these professionals work informally cleaning homes, apartments and businesses. Therefore, private employers and workers are covered by OSHA, but those who are self-employed are not [20]. This may impact immigrant women significantly, who rely on flexible work hours and informal work arrangements. For these women, the National Domestic Workers Alliance (NDWA) serves house cleaners, nannies and home care workers, advocating for worker rights, including safe and healthy work conditions [21]. NDWA offers resources specific to safe cleaning practices for self-employed cleaning professionals [22]. Those with low literacy may have challenges accessing this website-based information.

To date, little is known regarding the knowledge (via training experiences), attitudes and behaviors of Latinas who clean occupationally. This mixed methods study explores the knowledge, attitudes, and behaviors regarding occupational practices among Latinas from different countries of origin and acculturation levels, working in the cleaning industry. Findings may be utilized to create culturally tailored interventions for this underserved population.

## Methods

This was a convergent mixed methods study [23–25]. Due to the exploratory nature of this pilot study, power was not calculated. The qualitative focus groups were designed using semi-structured, open-ended guided questions and probes. The quantitative component was a closed-ended 43-question survey [26] on demographics and cleaning practices at work and home. The development of the questions for the focus groups and survey were guided by the Social Cognitive Theory (SCT), which addresses the interpersonal level and attributes human behavior to the continual interaction of personal factors, environmental influences, and personal behavior [27–29]. The focus group guide questions were designed to build upon and complement the information elicited by the survey. Both the focus group and survey questions were designed to be culturally and linguistically appropriate for the target population. They were created by the PI and then reviewed for cultural appropriateness and clarity by two co-authors and a Delphi panel of three faculty members.

### Research questions

Based on the stated purpose, the research questions are as follows: For Latina women from different countries of origin and acculturation levels who work in cleaning occupations in New Jersey (NJ):

RQ1: What is the *knowledge* base specific to cleaning procedures (via *training experiences)*?

RQ2: What are the *attitudes* regarding the presence of chemicals in cleaning products?

RQ3: What are the *cleaning routines (behaviors)*?

RQ4: What are the *cleaning products used (behaviors)*?

The study was conducted according to the International Conference on Harmonization (ICH), Good Clinical Practice (GCP), the Declaration of Helsinki, Institutional Review Boards (IRB) and in accordance with the U.S. Code of Federal Regulations on Protection of Human Rights (21 CFR 50) and was approved by the Hackensack Meridian Health (HMH) Institutional Review Board (IRB) under Pro #2019–0015. Written informed consent was obtained from each participant prior to entering the study.

### Participant recruitment

Participants were recruited from English as a Second Language (ESL) classes offered to parents of students enrolled at Hackensack High School in Hackensack, NJ using flyers and phone calls between June 2019 and February 2020. The English version of the recruitment flyer was worded at a 4th grade level according to the Flesch-Kincaid scale, and translated into Spanish [30]. Women who attended the ESL classes who had agreed to be contacted, were called by the study team in order to present the study. Those interested were provided with the description of the study, and upon confirmation of eligibility, were invited to participate in the focus group. To facilitate recruitment, participants also provided the study team with contact information for a friend or family member who also cleaned occupationally and was potentially interested in participating.

The study was designed to explore the responses of a maximum of five focus groups or until saturation was reached. Eligibility criteria included: Latina women aged 18 and older who worked in a cleaning occupation, spoke Spanish and/or English, and were able and willing to provide written informed consent. To be eligible, participants had to be willing to participate in a 90-min focus group discussion, which included completing a paper and pencil survey exploring their knowledge, attitudes and behaviors specific to cleaning practices and perceptions on health needs.

### Data collection

Focus groups were conducted between July 2019 and February 2020, with a total of fifteen participants. The focus group topic guide questions were aligned with the research questions and constructs of the SCT. General topics covered included cleaning routines both at home and at work, purchasing behaviors and access/usability of cleaning products, and training experiences. Examples for focus group questions included:

- Tell me about the cleaning routine at your home in a regular week. Who does what / when?
- Tell me about how you first learned to clean at home.
- Tell me about the cleaning routine at work in a regular week. Who does what / when?
- Tell me about how you first learned to clean at work.

Additionally, probing questions were used to elicit more detail during discussions and to clarify responses [31].

Focus groups were conducted in Spanish with a bilingual facilitator and a notetaker. Each focus group discussion lasted up to 45 min, followed by a written paper and pencil, interviewer-administered survey which took approximately 15–20 min for a total of up to 90 min for each session, including the informed consent process. The method for translating the focus group guide and survey into Spanish followed the U.S. Census Bureau’s *committee approach* (consensus method) for translating data collection instruments and supporting materials [32].

All participants received a $25 gift card for their time and were provided light refreshments. After the focus group discussion and surveys were complete, participants were provided with a fact sheet on safer cleaning. This educational flyer was published by government agencies (CDC, NIOSH and OSHA) in Spanish and English [33]. Both language versions were available at the focus groups.

In focus group (FG) #1 (pilot data), literacy issues and unfamiliarity with survey participation was a barrier to correctly and adequately filling out the survey. With IRB approval, three bilingual survey assistants were added, starting with FG #2 to provide one-on-one assistance with reading and filling out the survey. Only survey data from FG #2 and #3 are presented in the study results (*n* = 9). The survey assistants were all from Latin American countries and spoke Spanish as their first language. They did not provide translation, as the survey was already translated for the participants. It must be noted that several survey questions allowed for multiple answers to be selected (such as what PPE is worn). Thus, frequency responses may be greater than 9.

### Data Analysis

#### Transcriptions

The audio recordings of each session were downloaded and sent for translation. As all three focus groups took place in Spanish, a certified bilingual translator transcribed the sessions verbatim into English for analysis. Translations were reviewed after the pilot phase (FG #1 and #2) and again after Focus Group #3. Saturation was reached after FG #3. The decision was made by the first author in consultation with the second author. The areas of similarity among the transcripts included: types of PPE used; lack of and inconsistency in employers providing PPE; products used occupationally and at home; factors involved in product selection (cost and scent); issues with product labeling (labels in English, not in Spanish); receiving little or no occupational safety training; learning how to clean at home during childhood.

#### Coding

Translated transcripts were read multiple times to ensure a general understanding prior to coding [24, 34]. The principal investigator (PI) then used Saldana’s color-coding method to organize the text by major categories [35]. The PI assigned emergent codes and sub-codes using in vivo and descriptive coding [24, 35]. The PI used Saldana’s two-part coding process of decoding to determine the core meaning of a passage and encoding to determine which code to use and label the passage [35]. Codes were reviewed by a senior researcher on the team for accuracy and met intercoder agreement of 80–90% recommended by Saldana [35].

Themes were determined inductively by reviewing the results of coding the data, to determine the main topics, or themes recurring in the translated transcripts from the three focus group sessions. Themes were used to further analyze and synthesize the data to form conclusions about the knowledge, attitudes and behaviors of the participants. The coding process and resulting codes, sub-codes, categories, and thematic analysis were used to interpret the data as well as inform the discussion and conclusions [24]. For the quantitative analysis, means, frequencies, and percentages were calculated for each of the survey questions.

## Results

It was determined that saturation was reached after three focus groups, totalling 15 participants. The qualitative data resulted in six themes. The quantitative data captured key demographic characteristics as well as knowledge, attitudes and behaviors information secured from the 43-question survey. The data reported here includes a subset of nine participants who provided this additional information, as those in the first focus group experienced challenges with survey participation and literacy barriers that were addressed in focus groups 2 and 3.

The qualitative and quantitative results of this convergent mixed methods study were analyzed separately, and then discussed together as suggested by Fetters, Curry and Creswell [36]. Quantitative survey data is presented first, followed by the qualitative data.

### Quantitative survey data

#### Social/demographic characteristics of participants

Participants’ country of origin included five countries: Ecuador (*n* = 4, 44.4%), Dominican Republic (*n* = 2, 22.2%), Mexico (*n* = 1, 11.1%), Peru (*n* = 1, 11.1%), and El Salvador (*n* = 1, 11.1%). Ages ranged from 41 to 61, with a median of 46.0 and a mean of 48.78 (standard deviation 6.72).

The number of years that participants lived in the U.S. varied from 8 to 31. The most frequent answer was 10–20 years (*n* = 4, 44.4%). The median was 17.0 years and the mean was 18.78 with a standard deviation of 7.85 years. All nine survey respondents were first generation immigrants to the U.S., meaning that they were born outside the U.S. Total household income (before taxes) were reported as *n* = 2 (22.2%) for each of the categories $10,000-19,000, $20,000–29,000 and $30,000–39,000 and *n* = 1 (11.1%) for each of the responses $50,000-59,000, $60,000–69,000 and $80,000 or more.

Lastly, the Social/Demographic section asked participants about their preferred language in a variety of communication scenarios, as summarized in Table 1. These six questions were included in order to determine the most appropriate language for designing a future intervention for this population. The response choices for each were: only Spanish, more Spanish than English, both equally, more English than Spanish, and only English.

**Table 1:** Demographics

Characteristic	Number of participants (*n* = 9)
Age range of participants	
40–45 years	3 (33.3%)
46–50	3 (33.3%)
51–60	2 (22.2%)
61–70	1 (11.1%)
Marital status	
Married or cohabitating	5 (55.6%)
Divorced/separated	3 (33.3%)
Never been married	1 (11.1%)
Highest level of education completed	
Elementary school (5th grade)	3 (33.3%)
Junior high/middle school (8th grade)	1 (11.1%)
High school or equivalent (12th grade)	2 (22.2%)
Technical school	1 (11.1%)
Bachelor’s degree (4 year college)	2 (22.2%)
What language(s) do you read and speak?	
Only Spanish	3 (33.3%)
More Spanish than English	6 (66.7%)
What language(s) do you speak at home?	
Only Spanish	4 (44.4%)
More Spanish than English	4 (44.4%)
Both equally	1 (11.1%)
In what language do you think?	
Only Spanish	7 (77.8%)
More Spanish than English	2 (22.2%)
What language do you speak with your friends?	
Only Spanish	6 (66.7%)
More Spanish than English	2 (22.2%)
More English than Spanish	1 (11.1%)

When asked “How long have you been in the cleaning profession?” (*n* = 9), five participants (55.6%) responded ten years or more, suggesting a high level of experience gained on the job. One participant each (11.1%) responded less than one year, 1–3 years, 4–6 years and 7–9 years. When asked “What type of job training would be helpful to learn new skills?” (*N* = 5) three participants (60.0%) preferred “Text messages to phone with tips and links to website resources.” One (20.0%) chose “Someone training me at my workplace” and two (40.0%) answered “Someone training me at a community center.” For Focus Group 3, the question was revised to gather more detail: “How would you like to receive training about health and safety practices related to your cleaning job?” The response options were increased to seven, which were answered according to the following (*N* = 4): Training provided through email = 1 (25.0%), Training provided through text messaging to your phone = 3 (75.0%), Onsite in-person training at workplace = 2 (50.0%), Onsite in-person training at community center = 2 (50.0%). The responses Not sure, Not interested in learning new skills at this time and Other (please explain) all received zero (0.0%). Cleaning locations and hours per week are summarized in Table 2.

**Table 2:** Cleaning location and hours per week

Type of place for occupational cleaning + hours per week	*N* = 9 (%)
# of units cleaned per week: Apartments	*N* = 7 (77.8%)
1–4 per week	6 (85.7%)
9–12 per week	1 (14.3%)
# of units cleaned per week: Homes	*N* = 6
1–4 per week	4 (66.7%)
9–12 per week	2 (33.3%)
# of units cleaned per week: Office Rooms	*N* = 3
1–4 per week	3 (100%)
# of units cleaned per week: Schools/daycares	*N* = 3
1–4 per week	3 (100.0%)
# of units cleaned per week: hotel/motel room(s)	0
# of units cleaned per week: restaurant(s)	0
# of units cleaned per week: factory(s)	0
other	0
# of hours spent cleaning for work	
1–20 h	3 (33.3%)
21–40 h	4 (44.4%)

All participants conducted a variety of cleaning operations within their occupational role, as shown in Table 3, with most participants spending 1–2 h per task category. Participants were also asked to specify the products used, which provides information regarding potential environmental exposures.

**Table 3:** Occupational cleaning activity, number of hours per week and products used

Activity	*N* = 9 (%)
Dusting	9 (100.0%)
1–2 h	6 (66.7%)
3–4 h	2 (22.2%)
5–8 h	1 (11.1%)
	Products used: Just use feather duster / and damp cloth 1/2 water and 1/2 disinfectant; “Pine Wall” (meant “Pinesol®”); cloth; Fabuloso®; lemon oil with paper towel; water and towel; wet towels and “doesn’t know.”
Mopping	8 (88.9%)
1–2 h	6 (75.0%)
3–4 h	2 (25.0%)
	Products used: 1/2 water and disinfectant / Fabuloso®; Bleach, Acid, Jaboven® powder; Mistolin®; Fabuloso® / Clorox®; Mr. Clean®; Fabuloso®; Fabuloso® and other products provided by my boss
Cleaning Bathrooms (e.g. bathtub, shower, toilet, sink)	9 (100.0%)
1–2 h	6 (66.7%)
3–4 h	3 (33.3%)
	Products used: “Cloro” (likely meant Clorox®) wipes one deep cleaning per week - using a spray and Ajax® for toilet bowl; Clorox® (2 responses), soap; Clorox®, Windex®, Mistolin®; Comet®, “Axax” (meant “Ajax®”) - toilets and Scrubbing Bubbles® - sink; Windex® (without ammonia) and Ajax®; Clorox® and Ajax®; Crema soft bleach / Ajax® powder, Windex® for mirror, Fabuloso®
Cleaning Kitchens (e.g. washing dishes, stove, sink)	8 (88.9%)
1–2 h	5 (62.5%)
3–4 h	2 (25.0%)
9 or more hours	1 (12.5%)
	Products used: Mr. Clean® Lemon / use fan; Palmolive® soap; Clorox® spray & wipes, spray degreaser for stove, Dove® dishwashing liquid; Easy off® spray (acid) [for] oven, Fantastic®; Easy-off® and Ajax® w/ sponge; Mr. Musculo® + vinegar + dish soap (Dawn®); For oven - Mrs. Musculo® / Stanley steel®; Orange citrus - to remove fat from the oven, “Down” (likely meant “Dawn®”) - fridge, Crema Soft Bleach
Cleaning Glass (e.g. windows, mirrors)	9 (100.0%)
1–2 h	9 (100.0%)
	Products used: Similar to Windex® / sometimes use alcohol; Windex® (5 responses); “Down” (likely meant “Dawn®”) & Windex®; Windex® / “Down” (“Dawn®”) soap.
Vacuuming	7 (77.8%)
1–2 h	1 (14.3%)
3–4 h	6 (85.7%)
	Products used: vacuum cleaner (3 responses); vacuum + broom
Sweeping	6 (66.7%)
1–2 h	6 (100.0%)
	Products used: no (none); broom (4 responses).
Laundry	2 (22.2%)
1–2 h	2 (100.0%)
	Products used: Oxiclean®, bleach, Downy®, Suavitel®; “Down” (likely “Dawn®”) soap for dishes (only wash towels that were used for cleaning).

Most participants had worked outside the cleaning industry. Six respondents indicated: Dunkin Donuts®; industrial machine handling classes, sewing and fashion design; Dunkin Donuts® (cashier), manufacturing [factory worker], printing office; taking care of kids; electronics [factory] - welding; plastic factory. Most participants (*n* = 9) were currently employed by a company either full time (*n* = 3) or part-time (*n* = 3) or were self-employed full time (*n* = 2) or part-time (*n* = 1), while one was employed seasonally.

When asked “How are you paid?” and for what location, seven participants (87.5%) responded by the hour (one indicating location was a school), one (12.5%) indicated being paid by the week, and zero chose by the day. One participant, not counted in the statistics, created her own response by writing in “by the house.” For the two respondents who also had second jobs, one reported being paid by the hour for cleaning apartments and another participant indicated being paid by the week. Additional characteristics of occupational cleaning activities are summarized in Table 4.

**Table 4:** Characteristics of occupational cleaning activities

Characteristic	*N* = 9 (%)
Person who purchases cleaning products used at work	
I purchase my own cleaning products (write-in cleaning place/location: “exclusive store cleaning products”)	1 (11.1%)
Boss or homeowner purchases them (cleaning place/locations: “House room,” “house,” “all”)	4 (44.4%)
The company provides the products (cleaning place/location: “school / apartments,” “does not know” and “apartment”)	4 (44.4%)
Other	0
Frequency of changing cleaning product brands at work	
never	4 (44.4%)
hardly ever (every few months)	4 (44.4%)
sometimes (monthly)	1 (11.1%)
all of the time (weekly)	0
most of the time (every few weeks)	0
Reason for choosing products	
The products are provided for me, so I don’t have a choice	7 (77.8%)
I choose what smells good	1 (11.1%)
I choose the products that I know work	2 (22.2%)
I choose based on the ingredients	1 (11.1%)
Other	1 (11.1%)
I choose based on price	0
All that apply	0
Cleaning with others	
people I met on the job	5 (55.6%)
friends	3 (33.3%)
no one else	3 (33.3%)
family members	0
kids under age 18	0
spouse/partner	0
other	0
Clean with (# of other people)	
1–4 other people	6 (66.7%)
none/alone	2 (22.2%)
5–10 other people	1 (11.1%)
11 or more other people	0

The participants use a variety of PPE. Most often, gloves were worn (88.9% of participants), followed by masks (66.7%), knee pads (33.3%), uniform (33.3%), special shoes or shoe covers (22.2%), and goggles or protective glasses (11.1%).

When asked “How long do you see yourself staying in this occupation?” four participants (44.4%) responded 1–3 years, and five (55.6%) indicated ten years or more. None indicated less than 1 year, 4–6 years or 7–9 years.

The survey responses regarding cleaning at home are summarized in Table 5.

**Table 5:** Characteristics of home cleaning activities

Characteristic	*N* = 9 (%)
Frequency of cleaning at home (other than to do dishes)	
daily	5 (55%)
weekly	4 (44%)
once a month	0
a few times per year/about once per season	0
Use of different cleaning products when seasons change (for example, using more disinfectant during flu season, etc.)	
no	8 (88.9%)
yes (specified Lysol® and hand sanitizer)	1 (11.1%)
Person who does most of the cleaning at home	
me	8 (88.9%)
relative	1 (11.1%)
friend	0
spouse or partner	0
kids (under age 18)	0
other	0
Person who purchases cleaning products used at home	
me	7 (77.8%)
family member (write-in: husband (1x), daughter (2x)	3 (33.3%)
friend	0
other	0
* one participant checked both myself and family members	

The focus group participants were asked to “Please write a list of five products you use at home (all-purpose cleaner, toilet cleaner, tub and tile cleaner, window cleaner, etc.).” The responses are organized by room, in Table 6.

**Table 6:** Products used at home

Kitchen	Floor	Windows/ Glass/ Mirrors	Bathroom	Other
vinegar (3x) bicarbonate soap Palmolive® Soap Downy®bleach 409®-grease Dawn®-grease degreaser-stove dish soap (Dawn®)-dishes vinegar-stove dove dish-dishes Mr. Clean®	Murphy Oil® Mistolin® (2x) vinegar (3x) baking soda Fabuloso® (3x) Pinesol®	Windex® (6x)	Clorox® (2x) Soft bleach Bleach Bobbles x clean bath vinegar (3x) Clorox® liquid baking soda degreaser (bathtub) bicarbonate (bathtub) vinegar (bathtub) span bubble (bathtub) dish soap (toilet) bleach (toilet bowl)	Oxiclean®-laundry Span Bubble-hand wash vinegar-crystals, marble vinegar-wall water-dust removal lemon oil-furniture (wood) Lysol®-air freshener Lysol®-surfaces sodium bicarbonate

### Qualitative data

#### Theme 1: knowledge of cleaning procedures via training experiences

A total of 15 participants participated in the focus groups and were included in qualitative analysis. Participants reported that their knowledge was gained through training experiences, starting at young ages by tradition, typically at home (“since I was a girl,” “at three years old”, “around seven years old” and “at thirteen”). Participants also reported knowledge was typically learned through observation (“she never taught me, I would watch them,” and “we Latinos learn to do things...without being taught”). There were also several comments about the lack of job training found in their professional careers. Two women in Focus Group 3 spoke about not being given any job training. One woman did say that “20 years ago when I started...some type of health person would come...they taught us how to take off the gloves...” This participant “liked that because...someone was concerned about us, who was interested in us.” Another participant commented that all she received were instructions on paper for her training: “the bosses only gave a paper when we started...and it says ‘training’ such and such product, and the instructions.” One participant pointed out the language barrier inherent with English instructions, that the paper that the boss read was in English, and were told, “that’s what it says, and sign here.” Another participant remarked that she has worked at her job for “19 years but they never gave me training.” Conversely, one participant was a trainer at her job where she had the opportunity to “teach them how to take care of themselves, how to protect themselves.” The dangers of working with chemicals was also discussed, “sometimes we don’t know how to use the products, and we mix them too.”

The predominant codes were: Started cleaning young, Tradition, Learned by observation. The category was: Experience drives knowledge, and the thematic analysis was: Knowledge gained through training experiences starts young by tradition and is typically learned through observation.

#### Theme 2: attitudes regarding the presence of chemicals in cleaning products

For attitudes, participants reported that product performance matters when making sure a space looks clean (“use some strong liquids, and it’s for the people,” and “smells good”). Preferred products include those that are more natural (“practically try to avoid everything … that’s why I use without air, without water … and bleach”) while another participant expressed doubts about natural products (“all organic things have chemicals too”). Preferred products come from reading about what works and word-of-mouth recommendations. Several participants reported not reading labels (“we don’t look [at labels]”) due to lack of time, already knowing what product to use, and most product labels being in English and difficult to read (“and they’re all miniscule … you’ve got to use a magnifying glass.”).

The predominant codes were: Looks & smells good, Word of mouth, Rarely read labels. The category was: Product performance matters and the thematic analysis was: Chemicals aren’t typically a consideration; Need to use what’s necessary to perform the job well.

#### Theme 3: cleaning routines (behaviors)

For behaviors, participants reported cleaning their own homes daily (“every day”) with an emphasis on regular cleaning routines. The importance of certain spaces was also expressed (“kitchen and bathroom are the main thing”) and two participants expressed that the kitchen is of prime importance, for when visitors are over. Occupational cleaning was heavy duty and may occur in a variety of locations (“apartments, houses, offices, and apartments after construction”). It includes tasks such as daily sweeping, mopping and cleaning windows as well as elevator doors, kitchens, student rooms (dorms), small cafeterias and offices at a university. The work is physically intense (“men’s work, the women do it,” “force ourselves to do the work,” “using heavy machines, the shampoo machine,” and “she does heavy duty work.”)

The predominant codes were: Clean daily, Flexibility, Heavy duty. The category was: Home and work routines and the thematic analysis was: Occupational cleaning involves physically intense work that extends from the job to home.

#### Theme 4: cleaning products used

For Cleaning Products Used, participants reported price as a key factor (buying products “at Costco®,” “at the supermarket … Costco® when there’s coupons,” “buy what’s more or less at a price that we can afford.”) Other Latinas reported being given products by their employer including several homeowners, a school and a university. Product names discussed included Fabuloso®, Windex®, Clorox®, dish soap, Easy Off® and Mr. Muscle®. For laundry, one participant reported “Tide® or Gain® laundry soap … fabric softener” while another mentioned “Suavitel®.” Sustainable options such as vinegar and water as well as bleach alternatives were also mentioned.

The predominant codes were: Price, what works, what’s provided.

The category was: Decisions on products used and the thematic analysis was: Use of cleaning products is driven by price and efficacy when given a choice.

#### Themes 5 & 6: home country & cultural identity

The topics of home country and cultural identity were discussed in response to an ice breaker question and carried through in some instances to the formal focus group discussions. Participants reported pride in being Latino (“Latino, something to be proud of!” and “my roots, my culture” as well as “we have that human warmth, full of love,” “we’re different”). The struggles of immigration and life both in participants’ country of origin and in the U.S. was also discussed (“we came to struggle … to this country,” “very hard working,” “I picked peanuts, peeled peanuts, tied tobacco”). Several participants discussed childhood conditions such as working in agriculture (“tend to the animals”) and growing up in a large family (“I have seven brothers,” “we’re a poor family,”) including one participant who was the first of 11 siblings and as a child went to school from 8 am to noon and then worked, including cooking for agricultural workers. Perceptions of Latinos in the workforce that were voiced in FG#3 included discussion of “people who don’t value work of Hispanic people” and in the U.S. “[it’s] all Hispanics working as cleaners.”

The predominant codes were: Proud**,** Large family, Loyal to roots back home, Worked hard**.** The category was: Home country & cultural identity and the thematic analysis was: Life in home country was hard and grounded by family ties.

## Discussion

Taken together, the qualitative and quantitative data show that training for cleaning typically began at a very young age by observation at home. Cleaning the home was expected, as well as helping in the community such as at church. Over half the participants have been cleaning for ten years or more. Several participants spoke of a lack of training and a desire to have training in person at their job, at a community center, or by a text intervention.

There were a variety of attitudes towards chemicals in cleaning products. Many participants felt it is important for spaces to “look good and smell good,” and therefore to use what will get the job done effectively. Participants have organized work groups where tasks are rotated and/or shared. Most (88.9%) are the primary person cleaning at home, which suggests increased exposure to cleaning chemicals beyond occupational exposures. Gloves and masks were the most frequently used PPE in both the survey and focus groups, but use is not consistent, and barriers exist such as cost, availability, and training for proper use. Under U.S. OSHA laws, employers must provide PPE free of charge to employees “when engineering, work practice, and administrative controls are not feasible or do not provide sufficient protection” [37]. Employers must also “ensure proper use” by training workers about best practices, including when and how to use PPE [37]. As previously mentioned, when cleaning professionals are self-employed, they are not covered by OSHA thus posing a potential problem for the use of best practices.

The data show a range of behaviors regarding product use. For participants who purchase their own products, price was a driving factor and some participants prefered traditional brands while others were interested in natural products. There was also an emphasis on “having to use” certain products, as dictated by the employer.

While the environmental health (EH) needs of this population are not explored herein, it is imperative that we recognize that reported EH impacts included respiratory and dermal issues, muscle strain, and breast cancer.

### The six constructs of the social cognitive theory as a lens

The SCT constructs (reciprocal determinism, behavioral capability, expectations, self-efficacy, observational learning (modeling) and reinforcements) proved instrumental as a lens through which to assess the study findings through the voices of the 15 Latina women who work in cleaning occupations. Bandura emphasizes “...people are self-organizing, proactive, self-regulating, and self-reflecting. They are contributors to their life circumstances, not just products of them” [38]. Their stories reflect Bandura’s constructs and his writings on SCT in cultural context, which reflected many of the participants’ life experiences via what Bandura terms a more “collectively oriented society” that is “highly communal” [27].

### Essential occupational cleaning workers

At the time this research was conducted, the world had not witnessed the extreme devastation of COVID-19, a pandemic that heavily impacted the Latino community [39]. As this pandemic emerged and continues to ravage communities on a global scale, the discussion of essential workers and of PPE use has undertaken an entirely new urgency. Those who clean houses, apartments, schools, daycares, and universities - as exemplified in this research - now help protect health in spaces both public and private. The unique vulnerabilities of minorities to COVID-19 coincide with the socio-economic barriers reported by participants in the present study [40–42]. Therefore, adequate protections on the job may serve to protect worker health from a variety of factors including chemicals and COVID-19. The importance of cleaning processes has been brought to a whole new level perhaps not experienced since the 1918 flu. The importance of training - and lack of consistent, uniform instruction on best practices in cleaning - has never been so urgent. The need for PPE, and the need for consistent, proper use remain some of the most pressing issues in public health.

This study lays the groundwork for a future intervention to empower Latinas in cleaning occupations - and employers across private and public sectors - to make cleaning training and PPE available regardless of a pandemic. As this study demonstrates, recruiting Latinas, a traditionally hard-to-reach group, is feasible when done in a culturally and linguistically appropriate manner. As described by Sheppard et al., ethnicity and language matching of research staff to subjects aided in approaching Latinas for study participation [43]. An intervention may be most effective via community-based participatory research (CBPR) that involves local stakeholders, Latina cleaning professionals and researchers collaborating together.

### Limitations

This research utilized non-probability purposive sampling. Therefore, the study has limited generalizability to other U.S. Latinas in the cleaning industry. Additionally, recruitment only from ESL classes for parents and their friends/family members at one NJ high school limited the number of study participants. Saturation was reached after three focus groups, where similar themes were repeated from the first and/or second focus group. A multi-site study including a larger number of women from a more diverse range of geographical areas would have allowed for comparisons to be made between locations. The survey could have also been distributed to Latinas in cleaning occupations who did not attend the focus groups, allowing for broader reach. While the focus groups were audio recorded, they were not video recorded, which increased the study team’s inability to accurately 100% of the time identify which participant was speaking. It must be noted that another possible limitation was social desirability bias from the participants, although the research team developed rapport to reduce potential bias. Finally, focus group attendance seemed affected by seasonal factors for participants without personal transportation who attended Focus Groups 2 and 3 in October 2019 and February 2020, respectively.

### Future research

This research study lays the foundation for a future intervention to empower Latinas in cleaning occupations towards a healthier work environment. As expressed in the focus groups and survey, participants have a desire for more training, preferably via a text-based intervention which will make it accessible on the job and at home. The intervention must be culturally and linguistically appropriate. This can be achieved via CBPR to engage members of the Latina cleaning community. An integrated partnership of community advocates, cleaning professionals and researchers will help create a comprehensive intervention to advance healthier occupational cleaning practices.

## Conclusion

This study was designed to provide a deeper understanding of the knowledge, attitudes and behaviors of Latinas in cleaning occupations. The findings will help in creating a future health intervention for this population.

Latinas in cleaning occupations in northern NJ face a range of social and health barriers including lack of training, inadequate PPE, and low literacy. The barriers found in this population are compounded by daily environmental exposures from occupational and home cleaning practices. To address these issues, the development of an intervention is warranted to provide training and resources for this critical population of essential workers.

## Data Availability

The datasets used for this study are available from the corresponding author on reasonable request.

## Abbreviations

CA: California
CBPR: community-based participatory research
ESL: English as a Second Language
FG: focus group
GCP: Good Clinical Practice
HMH: Hackensack Meridian Health
IRB: Institutional Review Board
NJ: New Jersey
PPE: personal protective equipment
PI: principal investigator
SCT: Social Cognitive Theory
